# Building Capacity in Monitoring Urban Liveability in Bangkok: Critical Success Factors and Reflections from a Multi-Sectoral, International Partnership

**DOI:** 10.3390/ijerph18147322

**Published:** 2021-07-08

**Authors:** Amanda Alderton, Kornsupha Nitvimol, Melanie Davern, Carl Higgs, Joana Correia, Iain Butterworth, Hannah Badland

**Affiliations:** 1 Centre for Urban Research, RMIT University, Melbourne, VIC 3000, Australia; melanie.davern@rmit.edu.au (M.D.); carl.higgs@rmit.edu.au (C.H.); joana.correia@rmit.edu.au (J.C.); iain@iainbutterworth.com (I.B.); hannah.badland@rmit.edu.au (H.B.); 2 Office of the Permanent Secretary, Bangkok Metropolitan Administration, Phranakhon, Bangkok 10200, Thailand; kkornkanit@gmail.com; 3 Melbourne School of Population and Global Health, University of Melbourne, Parkville, VIC 3010, Australia; 4 Iain Butterworth & Associates, Kyneton, VIC 3444, Australia

**Keywords:** sustainable development, low-to-middle income countries, social determinants, Thailand, global health, urban planning, capacity building, partnerships

## Abstract

Cities are widely recognised as important settings for promoting health. Nonetheless, making cities more liveable and supportive of health and wellbeing remains a challenge. Decision-makers’ capacity to use urban health evidence to create more liveable cities is fundamental to achieving these goals. This paper describes an international partnership designed to build capacity in using liveability indicators aligned with the UN Sustainable Development Goals (SDGs) and social determinants of health, in Bangkok, Thailand. The aim of this paper is to reflect on this partnership and outline factors critical to its success. Partners included the Bangkok Metropolitan Administration, the UN Global Compact—Cities Programme, the Victorian Government Department of Health and Human Services, the Victorian Health Promotion Foundation, and urban scholars based at an Australian university. Numerous critical success factors were identified, including having a bilingual liaison and champion, establishment of two active working groups in the Bangkok Metropolitan Administration, and incorporating a six-month hand-over period. Other successful outcomes included contextualising liveability for diverse contexts, providing opportunities for reciprocal learning and knowledge exchange, and informing a major Bangkok strategic urban planning initiative. Future partnerships should consider the strategies identified here to maximise the success and longevity of capacity-building partnerships.

## 1. Introduction

Recognition of the contribution made by urban environments to the health and wellbeing of city-dwellers is gaining momentum. It is now widely recognised by international agencies, such as the World Health Organization (WHO) and UN HABITAT [1,2,3,4]. Nonetheless, making cities more liveable, sustainable, supportive of health and wellbeing, and equitable remains a pressing issue. In addition to differences evident between cities and countries, health inequities within cities persist; these inequities are especially concerning in cities of low-to-middle income countries, which face additional challenges such as accelerated rates of urbanisation, informal housing, inadequate sanitation infrastructure, and inequitable access to health and social services [4,5]. At the same time, the prevalence of non-communicable diseases in low-to-middle income countries is rising, and emerging research links these health outcomes and related behaviours to features of urban environments [6]. In the coming decades, urban population growth will place additional pressure on cities located in low-to-middle income countries, particularly in Africa and Asia, where 90% of the growth in urban populations is projected to occur [7,8]. The rapid expansion of cities therefore presents both a challenge and an opportunity to plan and design cities that promote more equitable and sustainable outcomes [1].

To enable cities to meet current and future urban challenges and opportunities, several frameworks have emerged that bring together concepts of sustainability, health and wellbeing, and equity. The New Urban Agenda is a major contemporary global framework aimed at government, non-government organisations, and the private sector that establishes key commitments for sustainable and equitable urban development over the next two decades [9]. The adoption of this framework further reinforces the role of urban areas in achieving the UN Sustainable Development Goals (SDGs) [10], particularly their contribution to driving better health and environmental outcomes, as articulated in SDG 11 (Making cities inclusive, safe, resilient, and sustainable) and its supporting targets [11]. There is also growing policy interest in the concept of urban liveability as a feature of cities that promotes health and wellbeing [12]. Liveable cities have been defined as cities that are ‘safe, attractive, socially cohesive and inclusive, and environmentally sustainable, with affordable and diverse housing linked to employment, education, public open space, local shops, health and community services, and leisure and cultural opportunities, via convenient public transport, walking, and cycling infrastructure’ [13]. Previous work has contextualised this definition of liveability for cities in low-to-middle-income country contexts, recognising that a liveable city is also one where all residents enjoy clean air, access to clean drinking water and safe household fuels, adequate sanitation infrastructure, and safety from natural and climate-related disasters [14]. Liveability aligns closely with the SDGs and the New Urban Agenda, in that it contextualises these global goals to the local level [14], emphasising the need to address inequity alongside environmental sustainability and inclusive governance.

A key policy challenge for addressing health inequities within cities is that the health and wellbeing of city residents are shaped by decisions and policies that typically sit outside the remit of the health sector. These forces are commonly described as the social determinants of health, defined as ‘the conditions in which people are born, grow, work, live, and age, and the wider set of forces and systems shaping the conditions of daily life’ (WHO Commission on Social Determinants of Health, 2008). For example, provision of secure and adequate housing, transport infrastructure and systems, clean drinking water, and adequate sanitation all play a role in shaping health outcomes and inequities. However, responsibilities for delivering these social determinants sit largely outside of public health agencies and departments. The SDGs, and specifically SDG 17 (Revitalizing the Global Partnership for Sustainable Development), recognise that promoting and protecting urban health requires the formation of cross-sectoral partnerships that engage horizontally (i.e., across portfolios and sectors), vertically (i.e., across levels of government), and are viable across a variety of contexts. Emerging evidence shows that the potential for building these partnerships requires moving beyond fragmented ‘business-as-usual’ approaches. Instead, stakeholders need to come together to establish a shared framework for progress in cities, underpinned by principles of equity and sustainability [15].

Building policy and decision-makers’ knowledge of and capacity to create more equitable, liveable, and sustainable cities is fundamental to achieving the 17 SDGs [16]. The use of public health evidence to guide policy and decision-making outside of the health sector is finding traction globally. For example, in Australia, evidence-based urban health or ‘liveability’ indicators are gaining currency amongst policymakers, urban planners, economists, and local and state governments. These indicators are being used to identify, assess, and prioritise infrastructure needs and investment, targeting urban policy, and benchmarking and monitoring progress towards healthier, more prosperous and more equitable cities [17,18,19]. Beyond these practical uses, another benefit of developing policy-relevant liveability indicators is that they can be used to stimulate discussion amongst diverse actors, including civil society, not-for-profit organisations, the private sector, and various levels of government and government departments [20]. Hence, liveability indicators offer potential in developing capacity in both measuring and monitoring of urban development and as a mechanism for fostering connections across sectors and departments.

For cities in low-to-middle income countries, challenges in collecting and using disaggregated data related to urban health determinants have been identified as core issues to understanding and interrogating health inequities [4,14]. SDG 17 calls for capacity-building in collecting, developing, and using disaggregated data and metrics to drive sustainable development in low-to-middle income countries (targets 17.18 and 17.19). Yet, to-date, examples of urban health metrics that have been contextualised for cities in low-to-middle income countries—and case studies of their use in urban policy and practice—are lacking [7]. Examples are urgently needed demonstrating cross-sectoral partnerships that build capacity among, and exchange knowledge with, decision-makers in cities in low-to-middle income countries to not only improve health, but also reduce inequities. Such case studies help translate evidence about urban health and inequities into policy and practice across diverse city contexts. Importantly, partnerships based on the principles enumerated in SDG 17 provide a mechanism to stimulate knowledge exchange between cities and sectors, providing valuable lessons for advancing the SDGs at a local level.

Partnerships focussed on the SDGs need not be limited to locally-based partnerships; indeed, international partnerships can facilitate exchanging knowledge between actors in diverse contexts. For example, bilateral city-to-city partnerships between cities in diverse contexts have emerged as a popular mechanism for stimulating knowledge exchange and capacity-building [21]. Cross-cultural partnerships have also emerged between universities and research institutions as a means of building local capacity for developing evidence-based policies that promote citizens’ health and wellbeing [22]. These international partnerships, when grounded in a framework that prioritises local understandings of urban health issues, offer potential to share knowledge that guides solutions to the complex challenges different cities face. Indeed, SDG 17 explicitly calls for north-south, south-south, and triangular partnerships (target 17.9) as a key mechanism for driving sustainable development, alongside the involvement of multiple stakeholders in knowledge sharing across diverse contexts (target 17.16).

Throughout the development of international partnerships and capacity-building initiatives, a key consideration is how to structure activities and engagement in a way that enables knowledge sharing, prioritises local knowledge, and stimulates cross-cultural awareness and reflection. Indeed, Archer and Dodman contend that ‘the way in which capacity building activities have been conceived of and implemented has seldom been examined to assess its broader implications’ [23] (p. 69). Critical reflections on the structuring of activities, implementation, and factors that enabled or hindered success are increasingly recognised as an important type of knowledge for advancing sustainable development. Termed ‘reflective praxis’, these reflections have been highlighted by scholars as essential for translating urban health research into meaningful changes in cities [24]. 

A growing body of evidence is emerging to address these gaps, providing examples of capacity-building initiatives and reflecting on their processes and outcomes. For example, several case studies from the Asian Cities Climate Change Resilience Network have provided an examination of capacity building activities in cities across India, Thailand, Vietnam, and Indonesia, reflecting on factors and conditions enabling or inhibiting their success [23,25,26,27]. These case studies highlight the importance of conceptualising knowledge exchange as a two-way, iterative process, rather than a unidirectional transfer from the ‘more knowledgeable’ to the ‘less knowledgeable’ [23]. Further, they identify several factors shaping the success of the partnerships, including the importance of local champions [23,27] and strategic commitment at the city, state, and national government levels [25,27]. Challenges for the long-term sustainability of capacity-building efforts, such as staff turnover [27], data availability, access, and quality [25,26], and dedicated financial support [25,27] have also been identified. These reflections and case studies can offer insightful direction for future capacity-building initiatives at the city level, informing the design of activities to maximise long-term impact.

### Study Aim

The aim of this paper is to reflect on experiences from an international partnership designed to build organisational capacity in using and interpreting liveability indicators, aligned with the SDGs and social determinants of health, in Bangkok, Thailand [28]. Here, we discuss factors that were critical to its success and reflect on lessons that could be applied to future partnerships. Specifically, this paper’s objectives are to:

- describe partner organisations’ perspectives on motivators and outcomes for engaging in an international partnership anchored in the SDGs;
- present a tangible example of an international partnership developed to build capacity in and facilitate reciprocal learning; and
- provide critical reflections and lessons from the partnership.

## 2. Materials and Methods

### 2.1. Theoretical Framework: Capacity-Building as Adult Learning

An ambition of this capacity-building partnership was to build the Bangkok Metropolitan Administration’s long-term capacity to use and maintain the indicators portal to support the monitoring of Bangkok’s 20-year Development Plan. A major premise of the capacity-building initiatives was that those living and working in a given context are best suited to define the issues most relevant to the setting [29], and therefore that the Bangkok Metropolitan Administration should play an active role in identifying capacity-building priorities. Further, this capacity-building partnership was anchored in the principles of adult learning, namely, supporting people ‘to make sense of and act upon the personal, social, occupational and political environment in which they live’ [30]. Drawing on Brookfield [30] and Foley’s [31] criteria for effective adult learning, capacity-building activities were designed to engage participants in a ‘purposeful exploration’ of liveability concepts, enable Bangkok Metropolitan Administration leaders to develop ongoing liveability strategies, examine alignment between liveability indicators and existing strategic plans, and support the use of the indicators portal in practice. Local knowledge and priorities for capacity-building were sought from the outset; this enabled ‘beginning where people were at’—identifying and using learners’ initial understanding and skills as a baseline for building capacity [30,31]. Bangkok Metropolitan Administration training priorities were identified by the Field Action and Strategic Action working groups, with additional input from the Strategy and Evaluation Department.

### 2.2. Study Design: Research Reflection

This paper presents a critical reflection (‘reflective praxis,’ as outlined by Grant and Thompson [24]) on the partnership underpinning the capacity-building activities undertaken to enable the measuring and monitoring of liveability in Bangkok. This capacity-building partnership involved a multi-sectoral collaboration spanning two cities and countries (Melbourne, Australia; Bangkok, Thailand) between 2017 and 2020. The partnership itself is described in further detail in the following sections.

### 2.3. Documenting Collaboration Experiences and Anticipated Outcomes 

The reflections presented in this paper are based on the authors’ own reflections, as well as those of the partnership steering group and Bangkok Metropolitan Administration working groups. These reflections were based on ongoing feedback sourced across the partnership’s lifespan, including verbal communication (e.g., comments and feedback given in meetings and workshops) and short written reflections provided by partner organisation representatives at the conclusion of the project. These written reflections addressed the partnership’s successes and lessons learnt, alongside outcomes resulting from participation in this partnership. The written reflections were summarised and synthesised into major themes by two researchers (AA, HB). These themes were supplemented with the research team’s own reflections, notes and recollections from meetings and workshops. The initial draft of the paper presented here was led by two researchers based at RMIT University (AA, HB) and circulated to representatives from partner organisations for critical review.

### 2.4. Partnership Aims and Capacity-Building Outputs

**Figure 1:**
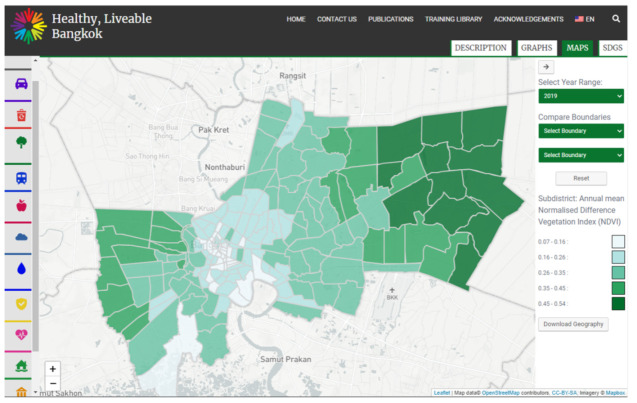
Interface of the partnership’s main product, a web-based liveability indicators portal. The interface allows Bangkok Metropolitan Administration officers to visualise liveability indicators mapped at the district or subdistrict (shown here) levels. Acknowledgements: Indicators site: RMIT University, 2021 [34]. Indicator data: Annual mean normalised difference vegetation index: Landsat-8 data courtesy of the U.S. Geological Survey, processed using Google Earth Engine. Subdistrict boundary data: Bangkok Metropolitan Administration (BangkokGIS). Retrieved 25 July 2019. Map data: OpenStreetMap contributors (https://www.openstreetmap.org/) (accessed on 7 July 2021), CC-BY-SA (https://creativecommons.org/licenses/by-sa/2.0/) (accessed on 7 July 2021). The Tracking Progress site uses Sparkjoy GeoC WordPress Theme version 1.9, Sparkjoy Studios, 2018. The indicators portal is housed by the International Institute of Sustainable Development’s Tracking Progress platform (https://www.tracking-progress.org/) (accessed on 7 July 2021). with site development provided by Sparkjoy Studios (https://sparkjoy.com/) (accessed on 7 July 2021). The map displayed in the image above was created using Leaflet (https://leafletjs.com/) (accessed on 7 July 2021) and imagery by Mapbox (https://www.mapbox.com/) (accessed on 7 July 2021).

The structure of the partnership, its outputs and processes, are described in detail elsewhere [14,15,28,32]. Briefly, this partnership sought to develop a suite of spatially-derived bilingual liveability indicators, aligned to various SDGs and social determinants of health, that could be used for measuring and monitoring liveability in Bangkok. This information was further adapted to guide the delivery of Bangkok’s Liveability Monitoring Framework and support the delivery of the 20-year Development Plan [33]. Spatial indicators were developed using both local and open-source spatial data. Where possible, these data were disaggregated by geography (i.e., units smaller than Bangkok-city level; typically district or sub-district scales) to enable local monitoring of health and liveability within Bangkok and identify inequities represented geographically. The main partnership output was a suite of spatial liveability indicators embedded in a bilingual liveability indicators portal site (hereafter termed ‘indicators portal’) housed by the International Institute of Sustainable Development’s Tracking Progress platform (https://www.tracking-progress.org/) (accessed on 7 July 2021). An example of the indicators portal interface is shown in Figure 1. At the conclusion of the capacity-building partnership in 2020, the Bangkok Metropolitan Administration assumed responsibility for maintaining the indicators portal into the future.

To support the ongoing use of the indicators portal by the Bangkok Metropolitan Administration, a library of training resources (e.g., pre-recorded training webinars, dataset, and software documentation) was developed by the team based at RMIT University. These resources covered topics of importance identified by the Bangkok Metropolitan Administration and partner organisations, including spatial and open source data acquisition, data cleaning, uploading and interpreting indicators, and conceptual underpinnings of the indicators (i.e., their alignment to the SDGs and importance for health and wellbeing) [14]. Originally planned for in-person workshops on-the-ground in Bangkok in 2020, these training activities were adapted to be delivered online due to travel restrictions during the global COVID-19 pandemic.

### 2.5. Partnering Organisations

#### 2.5.1. Bangkok Metropolitan Administration

The Bangkok Metropolitan Administration is the sole local authority responsible for the management of the city of Bangkok, Thailand. The Bangkok Metropolitan Administration’s 16 departments oversee a range of services including health; education; public works; strategy and evaluation; fire and rescue; drainage and sewerage; traffic and transport; culture, sports, and tourism; among others.

#### 2.5.2. Victorian Health Promotion Foundation

The Victorian Health Promotion Foundation (VicHealth) is the world’s first health promotion foundation, established by the 1987 Tobacco Act and funded by the State Government of Victoria (Australia), initially through dedicated government-collected cigarette taxes. Its central mandate is to promote good health and prevent chronic disease through innovative programming, research, and partnerships. An independent statutory authority with multi-partisan support, VicHealth works in partnership with governments, communities, and various organisations across a broad range of sectors.

#### 2.5.3. UN Global Compact—Cities Programme

The Cities Programme is the urban arm of the UN Global Compact, the world’s largest corporate sustainability initiative. The UN Global Compact takes a principles-led approach to corporate sustainability as enumerated in the Ten Principles, a set of commitments around fundamental responsibilities in the areas of human rights, labour, environmental sustainability, and anti-corruption [35]. As the urban arm of the UN Global Compact, the Cities Programme works to support a network of over 80 cities around the world to advance the SDGs and the Ten Principles of the Global Compact. The Cities Programme provides a platform for building cross-collaborations across private sector, civil society, local and regional governments to improve urban sustainability, resilience, and liveability. This is achieved through capacity-building programs, diagnostic tools, collaborative projects, and access to academic expertise. The motivator for Cities Programme involvement in this partnership was to facilitate dissemination of partnership findings to other Cities Programme member cities globally.

#### 2.5.4. Department of Health and Human Services (Victorian Government)

The Department of Health and Human Services is part of the State Government of Victoria (Australia). It oversees the development of policy, strategy, and service delivery across nine key policy areas: ageing; alcohol and drugs; ambulance services; children and families; disability; health and wellbeing; housing and homelessness; mental health; and public health. The Department of Health and Human Services held a strategic partnership with some of the authors (MD, HB) from 2011 to 2018 which included the provision of advice on relevant applications of partnership findings to the local Victorian context. More recently, the Department of Health and Human Services engaged with the Centre for Urban Research at RMIT University on mutual projects and research to enhance rural and urban liveability in the Victorian context. By partnering in this research, the Department of Health and Human Services sought to apply partnership findings to the Victorian context through engagement with Victorian local governments and regional partnerships, as well as further refinement of the liveability measures into Victorian state urban and health policies.

#### 2.5.5. Centre for Urban Research, RMIT University

The Centre for Urban Research, a multi-disciplinary research group located at RMIT University, a public university in Melbourne, Australia. Academics within the Centre have led and contributed to major programs of work conceptualising and operationalising liveability attributes and indicators through a social determinants of health lens.

### 2.6. Partnership Governance

Each partnering organisation nominated a representative to be part of the partnership steering committee. Representatives attended project meetings held every three months for the partnership’s duration, providing ongoing project guidance using a model of continuous improvement and reciprocal learning. At the start of the partnership, bilingual terms of reference were co-developed between the two lead organisations, Bangkok Metropolitan Administration and RMIT University. This was valuable for outlining and communicating the key roles of these two organisations. The governance structure was framed to focus on local knowledge and priorities at each stage of the partnership.

## 3. Results

### 3.1. Partnership Achievements within the Framework of SDG 17

This partnership targeted numerous SDGs, however, only the partnership-focused SDG 17 and related targets are discussed within this paper. Table 1 shows how the partnership’s activities and principles were designed to respond to several of the SDG 17 targets. The international, cross-sectoral nature of the partnership enabled particular outcomes to be achieved. First, the collaboration between partners based in high income (Australia) and upper-middle income (Thailand) country contexts was purposefully designed to stimulate two-way knowledge exchange around progressing and monitoring liveability and sustainable development (SDG 17.9, 17.16). A key outcome was the creation of a suite of spatially (geographically) disaggregated liveability indicators aligned to the SDGs and contextualised for Bangkok (SDG 17.18) and resources to support their ongoing use, updating, and interpretation (SDG 17.19). The authors expect that this also has relevance, and potential scalability, across other cities in the region and in low-to-middle income countries further afield (SDG 17.16, 17.18).

### 3.2. Contextualising Liveability in Diverse Contexts

The concept of liveability and applying it across diverse contexts was of interest for all partnering organisations. For example, liveability was already a feature in each partner’s strategic plans (e.g., 2015–2019 Victorian Public Health and Wellbeing Plan) or existing work programs [12,17,36,37,38]. Similarly, strong commitment to progressing the SDGs and targets was reported across partnering organisations as being important for framing this partnership. Shared challenges related to management of urbanisation and population growth. For Melbourne-based partners, understanding how liveability concepts could be contextualised and applied to settings other than metropolitan Australia was important. When the partnership commenced in 2017, the Centre for Urban Research was in the process of developing spatial liveability indicators for Australia’s 21 largest cities, advancing previous work undertaken for Australia’s capital cities [39]. Partners identified that having indicators available beyond urban Australia, and developing the methods to construct these, were important outcomes for guiding their own work programs. Consequently, the methods developed through this capacity-building partnership directly informed the creation of spatial liveability indicators that were subsequently applied to regional cities of Victoria [40,41,42,43,44], and Australia [18]. In turn, these indicators have been used to inform VicHealth and Department of Health and Human Services strategies. From the Cities Programme perspective, this partnership provided a case study to share knowledge with other cities from middle-income countries that form part of the Cities Programme network. In addition, because this was the first in-depth liveability indicator partnership focusing on the SDGs and localised in Southeast Asia, information was also shared with a range of organisations working within the East Asia-Pacific region. These included Thai Health, The Asia Foundation, and the UN Centre for Regional Development.

### 3.3. Opportunities for Reciprocal Learning and Knowledge Exchange

Partners were motivated by the potential for reciprocal learning and knowledge exchange opportunities across organisations and country contexts. To enable this, regular partner workshops and meetings held throughout the partnership identified present and future shared challenges and opportunities, regardless of geographic context. These included the pressure to deliver social infrastructure (e.g., schools, health and social services) to a growing urban population; increased vulnerability to floods, heat waves, and extreme weather due to the effects of climate change; the need for increased provision of quality, affordable housing; among others [45]. Partnership activities were designed with reciprocal learning and two-way knowledge sharing in mind; for example, several face-to-face visits were planned with workshops and roundtable discussions between partners to stimulate the sharing of knowledge and expertise.

### 3.4. Informing Strategic Planning in Bangkok

This capacity-building partnership was designed to strengthen the Bangkok Metropolitan Administration’s organisational capacity for progressing and monitoring Bangkok’s liveability, which would directly support Bangkok’s 20-year Development Plan [33]. Important capability development included strengthening expertise in sourcing, using, and interpreting spatial local and open-source data and indicators. The Field Action Working Group was responsible for overseeing and providing feedback on data sourcing, the development of the liveability indicators for Bangkok, and the online indicators portal. This group also considered mechanisms for the Bangkok Metropolitan Administration’s annual updating of spatial data into the future to allow for ongoing monitoring of liveability in Bangkok. The Strategic Action Working Group was responsible for knowledge exchange, enhancing understanding about Bangkok’s liveability indicators, and making policy recommendations to agencies and departments responsible for progressing Bangkok’s liveability strategies and initiatives. The Strategic Action Working Group is also charged with establishing practical guidelines to implement Phase 3 of the 20-year Development Plan (2023–2027) and coordinating the Bangkok Metropolitan Administration annual action plan from 2023 to 2027.

One of the outcomes stemming from this capacity-building partnership has been the prioritisation of the SDGs and the social determinants of health in the frameworks and metrics being explicitly used to monitor and evaluate Bangkok’s progress towards liveability. For example, Bangkok’s Liveability Monitoring Framework tracks Bangkok’s progress towards a more liveable city; this framework has been aligned with the 20-year Development Plan for Bangkok. The six themes of the Development Plan are fundamentally tied to Bangkok’s liveability and the indicators available in the portal. These themes are: a safe city; a comfortable, green city; a city for all; a compact city; a democratic city; and a city of economy and learning [33]. Strategic alignment between the liveability indicators developed in this partnership and the metrics and themes included in Bangkok’s 20-year Development Plan has been mapped by the Bangkok Metropolitan Administration. The Bangkok Metropolitan Administration is currently developing a policy proposal to formally incorporate the liveability indicators developed through the capacity-building partnership into Phase 3 of the 20-year Development Plan, to enable progress to be monitored against the liveability indicators. Further, it is envisioned that the liveability indicators can be used alongside other spatial indicators in the coming years to guide, measure, and monitor Bangkok’s long-term recovery from the social, economic, and environmental impacts of the COVID-19 pandemic.

## 4. Discussion

### 4.1. Lessons for Future Partnerships: Critical Operational Success Factors

Several factors were critical to the success of this capacity-building partnership. First, individuals and groups within the Bangkok Metropolitan Administration acted as champions of this partnership, and the urban liveability agenda more broadly. For example, the active engagement of a Strategic Division Director who acted as a dedicated bilingual liaison within the Bangkok Metropolitan Administration provided a critical point of contact between the partners in Melbourne and Bangkok. Her contributions included supporting regular communication between Bangkok- and Melbourne-based partners (both through email and attending and contributing to virtual meetings) and establishing a steering committee comprised of executives, as well as two working groups, within the Bangkok Metropolitan Administration. The first Bangkok Metropolitan Administration working group, known as the Field Action Working Group, advised on technical matters. The second working group, the Strategic Action Working Group, was responsible for translating and embedding findings from the capacity-building partnership into current and future Bangkok Metropolitan Administration strategies. In total, over 50 persons within the Bangkok Metropolitan Administration were involved, including the Governor of Bangkok, Chief Advisor to the Governor, Deputy Governor, Chief of Departments, and policy and planning analysts. This senior-level support for the partnership, along with the Governor of Bangkok’s endorsement of the development of the indicator portal, enabled the sustained engagement of the Bangkok Metropolitan Administration, even throughout the global COVID-19 pandemic. Previous works in other cities have found that this senior-level commitment is critical in providing resources and momentum for SDG projects and partnerships. Further, involvement of leadership and analysts across a broad range of departments encouraged a clear, consistent vision of liveability in Bangkok and acknowledgment of how different government departments and agencies contributed to this vision.

Second, the partnership governance structure of each partner having a representative on the partnership steering committee was of value. Each representative provided strategic advice to inform partnership and engagement activities in this partnership, while also being able to use findings in ‘real-time’ to inform their own work. Taking the example of the Cities Programme, a Project Development Manager worked to embed partnership findings into ongoing initiatives by refining the Cities Programme’s CityScan-VLR, a diagnostic tool that provides a framework to support local governments in developing a coherent and systematic assessment of SDGs, and understanding how to report and prioritise actions into an implementation roadmap.

Third, a commitment to co-production of the indicators with the Bangkok Metropolitan Administration, with ongoing review by the Field Action and Strategic Action working groups, was critical to enhancing the relevance of the liveability indicators to Bangkok’s local policy and planning context. Rather than being a linear process, development of liveability indicators was done iteratively. Each review of the working list of indicators and proposed data sources stimulated urban capability training and development, such as strengthening expertise in sourcing, using, and interpreting spatial local and open-source data and indicators, and aligning liveability indicators and outcomes to the SDGs. Further, feedback from the Bangkok Metropolitan Administration was that training activities fed back into indicator refinement. For example, one training resource stepped through the process of data sourcing and mapping a liveability indicator using an open-source geographic information system software, QGIS [46]. This training resource was highlighted by the Bangkok Metropolitan Administration as being particularly useful, as it stimulated deeper understanding of existing data sources, leading to the acquisition of additional spatial data.

Fourth, consideration of how to best support the ongoing use of the indicators portal and longevity of organisational capacity from the outset was key. This included incorporating a six-month handover period to the Bangkok Metropolitan Administration within the capacity-building program design and future-proofing the training resources provided (e.g., recorded webinars and training videos that can be accessed as required).

### 4.2. Lessons from This Partnership for a Post-COVID-19 Context

This capacity-building partnership had to be rapidly adapted in response to the COVID-19 pandemic, providing lessons that will be potentially relevant for future partnerships in a post-COVID-19 context. First, using a flexible range of engagement strategies (face-to-face visits, development of online resources) strengthened relationships between partners and improved partnership outcomes. We were fortunate to have had face-to-face meetings in 2017, 2018, and 2019 with all partners, which proved highly valuable in sharing knowledge, expertise, and building trust between partners. However, two additional face-to-face visits scheduled for 2020 in Bangkok were cancelled due to COVID-19 travel restrictions. Instead, these activities were conducted online. We found that providing recorded online resources (e.g., training webinars) were invaluable to the longevity of capacity-building efforts. By making these resources available into the future, it is anticipated this will help reduce the impact of personnel changes on long-term organisational capacity and reach a wider audience of current and future end-users of the indicators portal. We recommend partnerships and capacity-building projects in future to consider how activities can be structured using a range of flexible formats, including both face-to-face visits and a library of stable online resources.

As a consequence of the COVID-19 pandemic, there were personnel changes throughout the partnership, including resources and representatives of our partner organisations being reassigned to other duties. This resulted in several partner organisations being unable to contribute to the level originally planned, and some representative turn-over within partner organisations. Further, in light of shifting priorities, the UN Global Compact and RMIT University terminated their joint initiative, the Cities Programme, in early 2021. We learned, belatedly, that our terms of reference had not provided all partners with clear guidelines for how to respond to these scenarios. While a bilingual terms of reference document had been co-developed between the Bangkok Metropolitan Administration and RMIT University, we would suggest future partnerships include all partner organisations in this process.

## 5. Conclusions

This partnership provides a tangible example of capacity-building, involving governments, academia, and not-for-profit organisations working across two diverse city contexts, with relevance for future international partnerships around the SDGs. Tangible partnership outputs include strengthening expertise in sourcing, using, and interpreting spatial local and open-source data and indicators, housed in a web-based indicators portal and supported with a library of stable online training resources. Furthermore, the Bangkok Metropolitan Administration developed Bangkok’s Liveability Monitoring Framework, aligned with the 20-year Development Plan, for tracking Bangkok’s progress towards a more liveable city. This framework was supported by the liveability indicators developed through this partnership. Beyond this, the potential for reciprocal learning and knowledge exchange opportunities across organisations and country contexts was stimulated through this partnership.

Numerous critical operational success factors were identified that could inform future international partnerships around the SDGs. These included having a bilingual liaison and champion based in Bangkok, establishment of two active working groups in the Bangkok Metropolitan Administration, a commitment to co-production of the indicators, and incorporating a six-month hand-over period. Other successful outcomes from the partnership included contextualising liveability in diverse contexts, providing opportunities for reciprocal learning and knowledge exchange, and the partnership aligning to and informing a major Bangkok strategic urban planning initiative. From the outset, future partnerships should seek to establish a shared framework anchored in the SDGs and consider what strategies are needed to support the longevity of capacity-building outcomes and seek to align capacity-building efforts and tools with local policies and strategies.

## Figures and Tables

**Table 1:** Partnership activities and principles in the context of SDG 17 targets.

SDG 17 Target	Description of the SDG 17 Target	Partnership Activities and Principles
17.9	‘Enhance international support for implementing effective and targeted capacity-building in developing countries to support national plans to implement all the sustainable development goals, including through North-South, South-South and triangular cooperation.’	Involve partners across diverse sectors (public health, urban planning), organisations (local and state governments, not-for-profit organisations, academia), and country contexts (Thailand, Australia) Capacity-building priorities and needs determined by Bangkok Metropolitan Administration, rather than externally-driven
17.16	‘Enhance the global partnership for sustainable development, complemented by multi-stakeholder partnerships that mobilize and share knowledge, expertise, technology and financial resources, to support the achievement of the sustainable development goals in all countries, in particular developing countries.’	Face-to-face visits with knowledge-sharing workshops and roundtable discussions between partners integrated into capacity-building program design Bangkok Metropolitan Administration-led site visits in Bangkok (cancelled due to COVID-19 pandemic) Documentation to support replication of methods and ongoing indicator updates by Bangkok Metropolitan Administration Library of online training resources (webinars) to build capacity in using, interpreting spatial liveability indicators aligned to SDGs Indicators disseminated through International Institute of Sustainable Development’s Tracking Progress portal, contributing to international knowledge exchange around liveability and SDGs
17.18	‘By 2020, enhance capacity-building support to developing countries, including for least developed countries and small island developing States, to increase significantly the availability of high-quality, timely and reliable data disaggregated by income, gender, age, race, ethnicity, migratory status, disability, geographic location and other characteristics relevant in national contexts.’	Indicators portal housing a suite of SDG-aligned, spatially (geographically) disaggregated liveability indicators Indicators developed using both local and open-source data, with potential to be replicated and applied to other cities in low-to-middle income countries Six-month handover period incorporated into capacity-building program design from the outset to support long-term capacity-building
17.19	‘By 2030, build on existing initiatives to develop measurements of progress on sustainable development that complement gross domestic product, and support statistical capacity-building in developing countries.’	Replicable liveability indicators aligned to the SDGs and social determinants of health and wellbeing Bangkok Metropolitan Administration policy proposal (in development) to embed liveability indicators as metrics in Bangkok’s 20-year Development Plan

Note: A full list of the SDG 17 targets [10] can be found at https://www.un.org/sustainabledevelopment/globalpartnerships/ (accessed on 2 August 2017).

## Data Availability

Not applicable.
